# Chinese Cerebrovascular Neurosurgery Society and Chinese Interventional & Hybrid Operation Society, of Chinese Stroke Association Clinical Practice Guidelines for Management of Brain Arteriovenous Malformations in Eloquent Areas

**DOI:** 10.3389/fneur.2021.651663

**Published:** 2021-06-09

**Authors:** Mingze Wang, Yuming Jiao, Chaofan Zeng, Chaoqi Zhang, Qiheng He, Yi Yang, Wenjun Tu, Hancheng Qiu, Huaizhang Shi, Dong Zhang, Dezhi Kang, Shuo Wang, A-li Liu, Weijian Jiang, Yong Cao, Jizong Zhao

**Affiliations:** ^1^Department of Neurosurgery, Beijing Tiantan Hospital, Capital Medical University, Beijing, China; ^2^China National Clinical Research Center for Neurological Diseases, Beijing, China; ^3^Center of Stroke, Beijing Institute for Brain Disorders, Beijing, China; ^4^Beijing Key Laboratory of Translational Medicine for Cerebrovascular Disease, Beijing, China; ^5^Department of Neurosurgery, The First Affiliated Hospital of Harbin Medical University, Harbin, China; ^6^Department of Neurosurgery, The First Affiliated Hospital of Fujian Medical University, Fuzhou, China; ^7^Gamma Knife Center, Beijing Neurosurgical Institute, Beijing, China; ^8^Department of Vascular Neurosurgery, Chinese People's Liberation Army Rocket Army Characteristic Medical Center, Beijing, China; ^9^Savaid Medical School, University of Chinese Academy of Sciences, Beijing, China

**Keywords:** assessment, brain arteriovenous malformation, eloquent area, guideline, treatment

## Abstract

**Aim:** The aim of this guideline is to present current and comprehensive recommendations for the management of brain arteriovenous malformations (bAVMs) located in eloquent areas.

**Methods:** An extended literature search on MEDLINE was performed between Jan 1970 and May 2020. Eloquence-related literature was further screened and interpreted in different subcategories of this guideline. The writing group discussed narrative text and recommendations through group meetings and online video conferences. Recommendations followed the Applying Classification of Recommendations and Level of Evidence proposed by the American Heart Association/American Stroke Association. Prerelease review of the draft guideline was performed by four expert peer reviewers and by the members of Chinese Stroke Association.

**Results:** In total, 809 out of 2,493 publications were identified to be related to eloquent structure or neurological functions of bAVMs. Three-hundred and forty-one publications were comprehensively interpreted and cited by this guideline. Evidence-based guidelines were presented for the clinical evaluation and treatment of bAVMs with eloquence involved. Topics focused on neuroanatomy of activated eloquent structure, functional neuroimaging, neurological assessment, indication, and recommendations of different therapeutic managements. Fifty-nine recommendations were summarized, including 20 in Class I, 30 in Class IIa, 9 in Class IIb, and 2 in Class III.

**Conclusions:** The management of eloquent bAVMs remains challenging. With the evolutionary understanding of eloquent areas, the guideline highlights the assessment of eloquent bAVMs, and a strategy for decision-making in the management of eloquent bAVMs.

## Introduction

Brain arteriovenous malformations (bAVMs) are an abnormal collection of blood vessels wherein arterial blood flows directly into draining veins without the normal interposed capillary beds, while no brain parenchyma is contained within the nidus. Brain AVMs may lead to spontaneous intracranial hemorrhage (ICH), seizures, neurological deficits, or headaches, usually in young people (1, 2). Current treatments, such as microsurgical resection, stereotactic radiotherapy (SRS), endovascular embolization, and multimodality treatments mainly aim at preventing hemorrhagic stroke (3). However, the risk of suboptimal outcomes must be carefully balanced between treatments and wait-and-see strategies. Several links remain unclear in the management of bAVMs, especially in those located in eloquent areas. Challenges exist in preoperative assessments of neurological function, prediction of operative risks, and decision-making of therapeutic strategy and method. The purpose of this guideline is to review current studies and develop recommendations for the management of bAVMs with eloquent areas involved.

## Methods

A multidisciplinary group was proposed by the Chinese Cerebrovascular Neurosurgery Society (CVNS) and Chinese Interventional & Hybrid Operation Society (IHOS) of Chinese Stroke Association (CSA) and confirmed by CSA Executive committee, including the clinical researchers on microsurgery, endovascular neurosurgery, stereotactic radiosurgery, neuroradiology, and functional neuroimaging. Researchers in each field were screened for important conflicts of interest and assigned to the specific subcategory by a face-to-face meeting. These subcategories included anatomy of eloquent areas; preoperative neuroimaging, neurological assessment; neurosurgery, endovascular surgery, stereotactic radiosurgery, multimodality treatments, and conservative treatment. Each subcategory was led by at least one author.

The group identified all available literature related bAVMs and neurological functions in humans, following the practices of the Task Force on Practice Guidelines for literature searches published by the American Heart Association/American Stroke Association (AHA/ASA). Given the focus of therapeutic questions remaining in clinical practices, we performed systematic literature searches, guided by Applying Classification of Recommendations and Level of Evidence (Tables 1, 2) (4). As the eloquent bAVMs were seldom studied specifically, extended searches involved all bAVM-related literatures on MEDLINE (1970–May 2020), with (“arteriovenous malformations” [MeSH Terms] OR “arteriovenous malformations” [All Fields]) AND (“brain” [MeSH Terms] OR “Intracranial” [MeSH Terms] OR “cerebral” [MeSH Terms] OR “cerebellar” [MeSH Terms] OR “brain stem” [MeSH Terms] AND 1970/1/1:2020/5/31 [Date–Publication]). Publications irrelevant to eloquent bAVMs were excluded. Works of literature were further screened by different terms specified to each subcategory. Methodological filters were used to identify RCTs, meta-analyses, and systematic reviews.

**Table 1:**
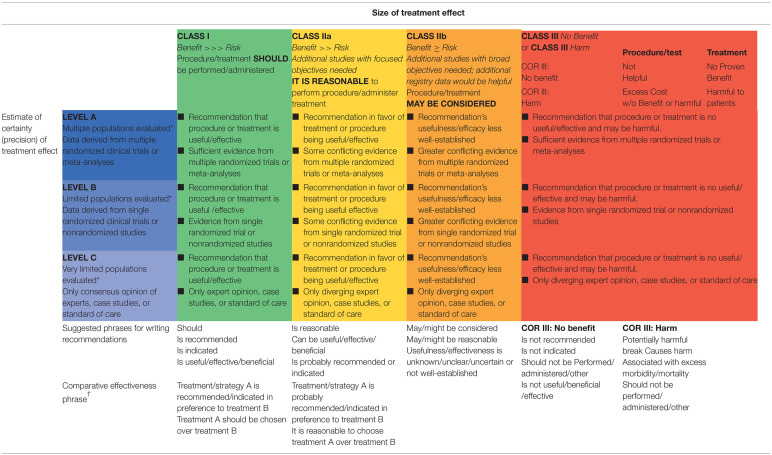
Applying classification of recommendations and level of evidence.

*A recommendation with Level of Evidence B or C does not imply that the recommendation is weak. Many important clinical questions addressed in the guidelines do not lend themselves to clinical trials. Although randomized trials are unavailable, there may be clinical consensus that a particular test or therapy is useful or effective*.

^*^Data available from clinical trials or registries about the usefulness/efficacy in different subpopulations.

*^†^For comparative effectiveness recommendations (Class I and IIa; Level of Evidence A and B only), studies that support the use of comparator verbs should involve direct comparisons of the treatment or strategies being evaluated. green: Benefit >>> Risk; orange: Benefit >> Risk; yellow: Benefit ≥ Risk; red: harm or no benefit*.

**Table 2:** Definition of classes and level of evidence used in recommendations.

**Class/level**	**Description**
Class I	Conditions for which there is evidence for and/or general agreement that the procedure or treatment is useful and effective
Class II	Conditions for which there is conflicting evidence and/or a divergence of opinion about the usefulness/efficacy of a procedure or treatment
Class IIa	The weight of evidence or opinion is in favor of the procedure or treatment
Class IIb	Usefulness/efficacy is less well-established by evidence or opinion
Class III	Conditions for which there is evidence and/or general agreement that the procedure or treatment is not useful/effective and in some cases may be harmful
Therapeutic recommendations	
Level of evidence A	Data derived from multiple randomized clinical trials or meta-analyses
Level of evidence B	Data derived from a single randomized trial or non-randomized studies
Level of evidence C	Data derived from a single randomized trial or non-randomized studies
Diagnostic recommendations	
Level of evidence A	Data derived from multiple prospective cohort studies using a reference standard applied by a masked evaluator
Level of evidence B	Data derived from a single grade A study or one or more case-control studies, or studies using a reference standard applied by an unmasked evaluator
Level of evidence C	Consensus opinion of experts

Drafts of recommendations were circulated to the entire writing group by online video conferences for feedback. Sections were revised and merged by the first authors. Comments of the merged draft were made by the entire writing group and got incorporated before the approval of the final draft. The corresponding authors revised the document in response to peer review. The manuscript was sent to the entire writing group again for additional suggestions and approval.

## Results

**Figure 1:**
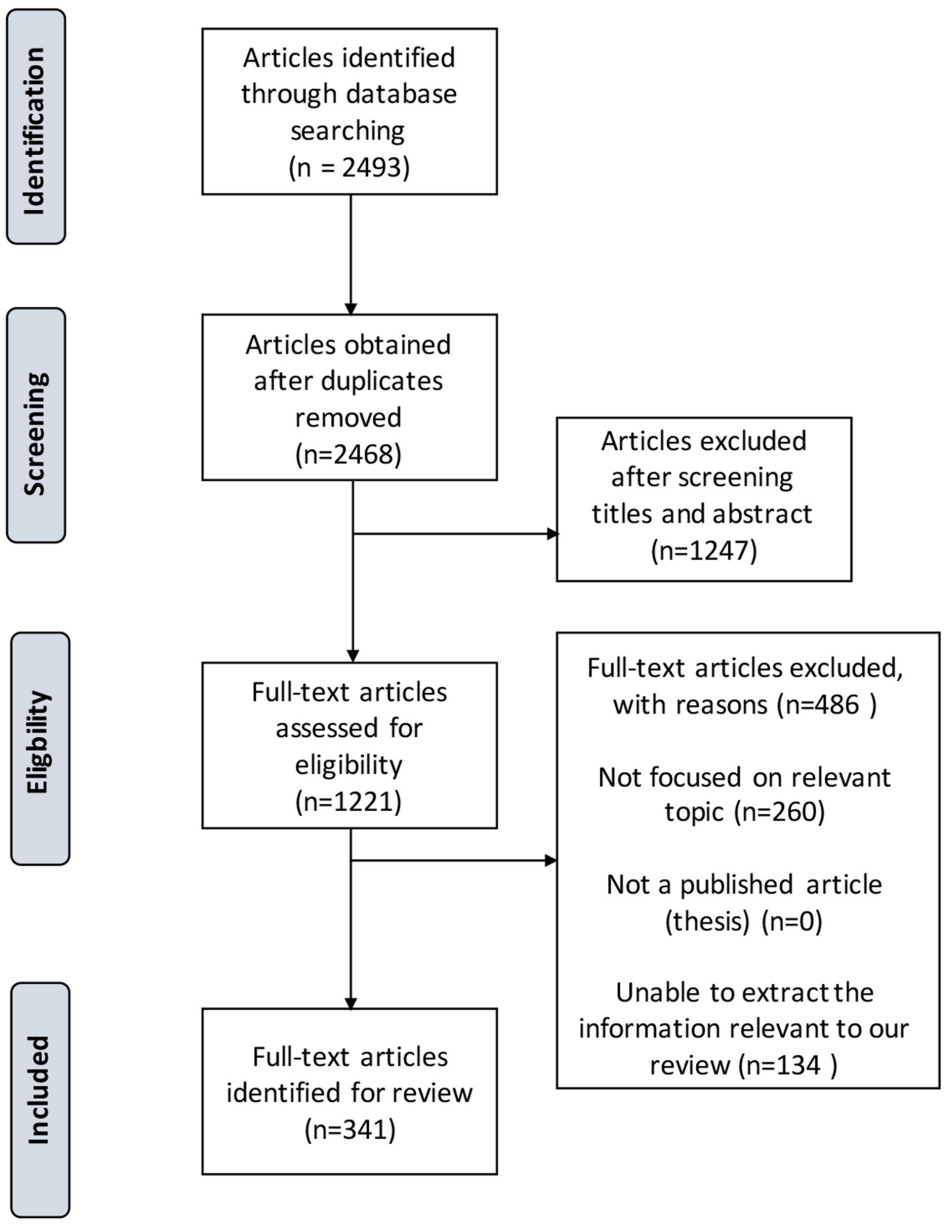
PRISMA study flow diagram demonstrating the number of articles retained at each stage of data acquisition.

A total of 2,493 bAVM-related results were obtained. Works of literature related to bAVMs, eloquent areas, neurological functions, and clinical techniques were identified. With in-depth interpretations, 341 pieces of literature were cited by this work, including 2 randomized clinical trials (RCTs), 8 meta-analyses, 224 clinical cohorts, 31 case reports or series, 46 reviews, 31 laboratory researches, and several literatures in other forms (Figure 1). The writing group summarized 61 recommendations for the management of eloquent bAVMs (refer to Table 3).

**Table 3:** The details of 55 recommendations.

**Level of evidence**	**Size of treatment effect**			
	**Class I**	**Class IIa**	**Class IIb**	**Class III**
A	1	0	0	1
B	18	16	3	1
C	1	14	6	0

### Anatomy of Eloquent Areas

#### Sensorimotor Brain Areas

Motor-related cortices mainly include: (1) primary motor cortex, located in the precentral gyrus and correspond to Brodmann's area 4; (2) supplementary motor cortex (SMC) which is in the medial side of the cerebral hemisphere and in front of the primary motor cortex, and is a major area related to motor programming and corresponds to the medial part of Brodmann's area 6; (3) premotor cortex (PMC) located in the lateral side of the frontal lobe, occupying part of the superior frontal gyrus, middle frontal gyrus, and precentral gyrus. The primary somatosensory cortex is in the postcentral gyrus, corresponding to Brodmann's area 1–3 and there is a corresponding relationship between areas in the primary somatosensory cortex and specific body areas. Moreover, the corticospinal tract (CST) is the most important fiber tract related to motor function, which consists of axons of pyramidal cells of the middle and upper part of the precentral gyrus and some other cortical regions.

#### Language Related Brain Area

##### Cortex

Language-related cortices mainly include (1) Broca's area: including the pars opercularis and the pars triangularis of the inferior frontal gyrus of the dominant hemisphere, corresponding to Brodmann's area 44 and the second half of Brodmann's area 45. Broca's area plays a vital role in the production of speech and understanding procedures (5, 6). (2) Wernicke's area: The Wernicke area is traditionally considered to be in the posterior third of the superior temporal gyrus (STG) of the dominant hemisphere (usually left hemisphere), corresponding to the rear of Brodmann's area 22 while there is no uniform definition of the specific range. Wernicke's area is mainly involved in the identification and understanding of speech. (3) Geschwind's area: The area is in the inferior parietal lobe of the left hemisphere, including the supramarginal gyrus and angular gyrus (Brodmann's area 39, 40). In recent years, brain functional imaging studies suggest that the Geschwind's area is an important language-related area and hub for multiple speech functions such as phonetic judgment, speech understanding, and reading (7–9). (4) Cerebellum: It was confirmed that the cerebellum is associated with logical reasoning and language processing (10).

##### Subcortical Fiber Bundle

The tractography and function of each language-related fiber bundle are still being studied. According to the anatomical position and function, they are currently divided into dorsal and ventral pathways. The dorsal pathway includes the arcuate fasciculus and the superior longitudinal fasciculus (11, 12). The ventral pathway includes the inferior fronto-occipital fasciculus, inferior longitudinal fasciculus, and the uncinate fasciculus connecting the temporal pole and the orbital gyrus (13). Previous studies suggest the dorsal pathway is mainly involved in the processing of phonetic functions while the ventral pathway is mainly involved in the processing of semantic functions. The theory is still being confirmed and remains controversial.

#### Vision Related Brain Area

It is located around the calcarine fissure of the occipital lobe. The cortex includes the primary visual cortex and extrastriate cortex. The primary visual cortex is in Brodmann's area 17 while the extrastriate cortex is in Brodmann's area 18–19. Optic radiation is the fiber bundle connecting the lateral geniculate body and the striate cortex (14). It starts from the lateral geniculate body bending backward around the temporal horn and trigone. Optic radiation can be divided into an anterior, middle, and posterior bundle. All of the bundles pass backward along the lateral wall of the occipital horn to the calcarine fissure.

#### Cognition Related Brain Area

At present, more and more attention is paid to cognitive functions. The hippocampus is an important cognition-related brain area called the “hippocampal region” which serves as a part of the limbic system (15). It is responsible for short-term memory, long-term memory, and spatial positioning. The anterior hippocampus is seen to be involved in decision-making under approach-avoidance conflict processing.

#### Other Function Related Brain Areas

Other eloquent areas include basal ganglia, thalamus, hypothalamus, brain stem, cerebellar peduncles, internal capsule, and deep cerebellar nuclei. Basal ganglia are associated with control of voluntary motor movements, procedural learning, habit learning, eye movements, cognition, and emotion (16, 17). The thalamus regulates states of sleep and wakefulness and inputs from the retina and processes sensory information as well as relays it. The hypothalamus coordinates many hormones and behavioral circadian rhythms, regulates complex homeostatic mechanisms, and is associated with fear processing and social defense (15, 18, 19). The brain stem conducts all information relayed from the body to the cerebral, cerebellum, and vice versa must traverse the brain stem. It has integrative functions involved in cardiovascular system control, respiratory control, pain sensitivity control, alertness, awareness, and consciousness (20, 21). Cerebellar peduncles are widely believed to mediate visual and auditory reflexes (22, 23). The internal capsule contains frontopontine fibers, corticobulbar fibers, CSTs, sensory fibers from the body, and a few corticobulbar fibers. Temporopontine fibers, optic radiation, and auditory radiations are also included (24–27). Deep cerebellar nuclei are involved in basic circuitry work involving coordination and the precision of limb movements (22, 28).

#### Brain Connectome

In recent years, studies regarding cognitive neuroscience identify that there are complex brain networks that interact with each other to perform various functions. Researchers revealed that there is a relationship between many neuropsychiatric diseases (such as Alzheimer's disease and schizophrenia) and the abnormal topological change in brain structural and functional networks. These studies provide us with a new approach to studying the pathological mechanism of BAVMs and of evaluating the surgical outcomes preoperatively.

### Preoperative Imaging Assessment

#### Routine Imaging Examination

(1) T1/T2 weighted Magnetic Resonance Imaging (MRI): to identify the anatomical location, range, and edema around the lesion. (2) Magnetic resonance (arterial) angiography (TOF-MRA): to demonstrate cerebral vessels and the surrounding brain tissue; to assist the comprehensive evaluation of nidus size, location, diffuseness, hemorrhage, feeding arteries, draining veins, and surrounding normal blood vessels (29). (3) Computed Tomography (CT): to assess acute subarachnoid hemorrhage and hemorrhagic stroke with a sensitivity >90% (30). Although limitations exist when detecting bAVMs, some features relevant to vascular abnormalities could be revealed, including dilated or calcified vessels along the bleeding edge and increased density areas representing abnormal vascular clusters. CTA is more acclaimed for its decreased invasiveness, good spatial resolution, and higher inspection speed. However, limitations of CTA lie in the presence of ionizing radiation and metal artifacts. CTA has high sensitivity (83.6–100%) and specificity (77.2–100%) in detecting vascular abnormalities in patients with parenchymal hemorrhage and vascular abnormalities, which can be used for initial differential diagnosis of a spontaneous cerebral hemorrhage.

#### Digital Subtraction Angiography (DSA)

DSA is the reference standard for diagnosing bAVMs and provides detailed information about angio-architectures and hemodynamics through dynamic images (31). Those with suspected bAVMs *via* CT or MRI are suggested to perform DSA for further clarification.

#### Blood-Oxygen-Level Dependence Functional Magnetic Resonance Imaging (BOLD-fMRI)

BOLD-fMRI is a non-invasive, non-radioactive, repeatable technology with high temporal resolution and spatial resolution. Processed data of BOLD-fMRI could display an activation map of functional areas and support the localize the sensorimotor area, speech area, and hemispheric dominance before operation. BOLD-fMRI includes the task-based and the resting-state ones.

In task-based BOLD-fMRI, block-designed scan tasks are commonly used (32). (1) Tasks of the detection of motor area activation: Finger movement (or dorsiflexion and extension of the foot) module and the block module alternately. The sensorimotor area of hands and feet is positioned by finger stretching movements, specified sequence of finger contrapuntal movement, or foot dorsiflexion. The time of each task or block module is no <20 s in general and the interval of adjacent task modules must not be longer than 128 s. (2) Tasks of the detection of speech area activation: speech tasks and the block module alternately. Speech tasks usually use picture naming, vocabulary association, verb generation, sentence judgment, etc. The form of speech tasks can be selected by their educational level, language habits, and target area. The time of each task or block module is no <20 s in general and the interval of adjacent task modules must not be longer than 128 s.

In resting-state BOLD-fMRI, patients are required to be awake with their eyes closed (or look directly at the cross target) lying quietly when performing the scan. Images are being used in the study of the brain network (33).

#### Diffusion Tensor Imaging (DTI) and Fiber Bundle Tracking

Spatial images are obtained by calculating the anisotropy of water molecules, based on which fiber can be tracked. Magnetic resonance equipment in 3.0 Tesla is commonly used with the spin-echo diffusion-weighted EPI technology to collect the image. The voxel size is 2 mm^*^2 mm^*^2 mm for more than 12 directions and the scanning time is about 5 min. White fibers displayed by DTI include projection fibers (corticospinal tract, cortico-nuclear tract, and thalamus radiation), association fibers (arcuate fasciculus, superior longitudinal fasciculus, inferior longitudinal fasciculus, inferior fronto-occipital fasciculus, uncinate fasciculus, and frontal oblique fasciculus), and joint fibers (callosum) (34).

#### Other Magnetic Imaging Techniques

Magnetic resonance spectrum (MRS) can be used to identify microhemorrhage. Arterial spin labeling (ASL) sequence can be used to differentiate malformed blood vessel mass and surrounding single supply artery and perfusion (35). Combined with other advanced encoding methods and physiological data, the characteristics of the hemodynamics of arteries and veins can be evaluated.

#### Sonography

Both extracranial and transcranial/transnuchal duplex sonography have been reported to be used as a non-invasive method for the diagnosis of bAVMs. Distinctive hemodynamic features could be detected and even evaluated. Extracranial sonography detects bAVMs by identifying the time difference of contrast bolus arrival between the internal carotid artery and internal jugular vein, as known as global cerebral circulation time (CCT). Schreiber et al. (36) reported that the CCT was 7.5 ± 1.1 s in healthy volunteers, while much faster (about 1.5 s) in bAVMs patients. Transcranial color duplex Doppler (TCCD) is the advanced product of conventional transcranial Dopplar (TCD). Both TCCD and TCD could record the velocity and pulsatility parameters of intracranial vessels, and identify the hemodynamic changes induced by bAVMs (37). TCCD was proposed as a valuable non-invasive, harmless, low-cost, widely available method for the detection and follow-up of hemodynamic changes of AVMs, especially for pediatrics (38). However, the effect of TCCD in neurological functional assessment has never been reported.

### Neurological Assessments

Muscle strength scales, Karnofsky performance scale (KPS), and modified Rankin Scale (mRS) could be used to assess motor functions. Edinburgh Dominance Scale was used to judge the dominant hemisphere of speech. If the lesion is in the dominant hemisphere, a speech-related scale or West Aphasia Battery (WAB) should be used to determine the existence of aphasia and the type and severity of it. It is important to make sure there are no related diseases that may affect the evaluation before assessments, such as hearing impairments and pyramidal tract injuries, Common language assessment scales include the Chinese version of WAB, Aphasia Battery of Chinese (ABC), China Rehabilitation Research Center Aphasia Examination (CRRCAE), etc. For bAVMs with vision-related brain areas involved, routine visual field examination (*via* visual field analyzer) is recommended before treatment. For lesions with cognition-related brain areas involved, Mini-Mental State Examination (MMSE) and Montreal Cognitive Assessment (MoCA) are recommended. National Institutes of Health Stroke Scale (NIHSS) is required for comprehensive assessments as well (7).

### Selection of Patients

#### Classic Grading Systems for Microsurgery

##### Spetzler-Martin (SM) Grading Scale

SM Grading Scale is the most commonly used grading system by far. Results of CT/MRI and DSA can be used in this scale to estimate the risk of surgical resection on: (1) the maximum size of lesion (<3 cm = 1 point; 3–6 cm = 2 points; >6 cm = 3 points), (2) the relative position of the eloquent area (eloquent area = 1 point, else = 0 points), and (3) the type of draining veins (only superficial veins = 0 points; deep veins = 1 point) (39). According to the SM Grading Scale, eloquent brain areas mainly include the sensorimotor area, visual and speech cortices, basal ganglia, thalamus, hypothalamus, brain stem, cerebellar peduncles, internal capsule, and deep cerebellar nuclei. The types of drainage are divided into: (1) with the superficial involved only, such as cortical veins draining into the superficial sagittal sinus, transverse sinus; and (2) with the deep drainage involved, indicating any draining vein to inferior sagittal sinus, Galen vein, and straight sinus. It has been confirmed that the grading system is an accurate predictor of surgical risk with a lower risk of permanent neurological deficits in the low-grade group (grade 1–2) than the high-grade group (grade 4–5). Spetzler et al. (40) recommended that bAVMs be divided into 3 categories for individualized diagnosis and treatment, including Type A (grade 1–2): recommended for microsurgical treatment; Type B (grade 3): recommended for individualized multimodal treatment; and Type C (grade 4–5): angiographic follow-up is preferred, while surgical treatment is only performed when aggravation of neurological deficits, recurrent bleeding or other conditions occurs.

##### Supplemented Spetzler-Martin (SM-Supp. or Lawton-Young) Grade

A supplementary of the SM grading scale was proposed to improve its predictive ability on microsurgical outcome with the following variables: patient age (<20 years old = 1 point; 20–40 years old = 2 points, >40 years old = 3 points), unruptured presentation (yes = 1 point; no = 0 points), diffuse (yes = 1 point; no = 0 points). The ROC curve in studies showed that it is more accurate than the SM scale (41–43).

##### Functional Image-Based Grading Scales

**Safe Lesion-to-Eloquence Distance (LED):** Studies had reviewed the influence of lesions involved in white matter eloquent fiber tracts, such as the subcortical cortical spinal tract (CST), optic radiation (OR), and arcuate fasciculus (AF) concerning sensorimotor, speech, and visual functions on the prognosis of patients, confirming that LED is an important risk factor for short-term and long-term neurological dysfunction in patients (32). It confirmed an acceptable LED to be 5 mm, which is significant in accurately assessing the risk and type of postoperative neurological dysfunction.

**HDVL Grading System:** This system was proposed to remedy the insufficiency of the SM grading scale on assessing (sub-)cortical eloquent structures and their LEDs. Each letter of HDVL stands for hemorrhage, diffusion, vein, and LED, respectively (Table 4). The fMRI and DTI-based functional imaging information are integrated into the grading system (32), and the vascular architectures of the lesion are considered to assist SM Grading in the preoperative evaluation of bAVMs, which provides a more accurate prediction of the prognosis.

**Table 4:** HDVL grading system.

**Variables**		**Score^**∫**^**
Lesion-to-eloquence^*^ distance (LED)	>10 mm	1
	5–10 mm	2
	<5 mm	3
Diffuseness^†^	Yes	1
	No	0
Deep draining veins^‡^	Yes	1
	No	0
Hemorrhagic history	Yes	0
	No	1

∫ *Total score = LED + Diffuseness+ Deep draining veins + Preoperative hemorrhage*.

*HDVL scores 1–3: operation is recommended*.

*HDVL scores 4–6: individualized multimodal treatment or conservative management is recommended*.

* *Eloquent areas include: sensorimotor, speech, and visual function related brain area identified by fMRI and functional white matter fiber tracts reconstructed by DTI such as cortical spinal tract (CST), optic radiation (OR), and arcuate fasciculus (AF)*.

† *Diffuseness refers to the inclusion of normal brain tissue in the nidus*.

‡ *Deep draining veins refer to part (or all) of the draining veins flow into deep veins, such as internal cerebral veins (ICV), basilar veins or precentral cerebellar veins*.

#### Grading Scales for Radiosurgery and Endovascular Surgery

Stereotactic radiosurgery (SRS) and endovascular embolization are used as solitary therapeutic options (44). Several radiosurgical-based and endovascular-based grading scales had been proposed (45). Modified-RBAS was validated by comprehensive comparative analysis of different SRS-related bAVM grading scales (46). For endovascular treatment, Jin et al. (47) published the results of the validity assessment for Spetzler-Martin, Puerto Rico, Buffalo, and AVMES grading systems to predict various outcomes *via* a multicenter retrospective study. The Puerto Rico scale was finally revealed to be superior in predicting short-term and long-term procedural complications. None of the current grading scales for endovascular surgery or radiosurgery have been widespread. Further large-size studies are expected to develop a simple and efficient grading scale for predicting outcomes of these treatments.

##### Modified RBAS Score

Modified RBAS bAVM score = 0.1 × Volume + 0.02 × Age + 0.5 × Location; Location score was 1 for basal ganglia, thalamus, and brainstem, and 0 for the rest of the brain. The following cutoffs of the bAVM score were used to predict the declining outcome of patients undergoing SRS: ≤ 1, 1.01–1.50, 1.51–2.00, and >2, with a score ≤ 1 predicting a 90% chance of lesion obliteration with no neurological decline.

##### Puerto Rico Scale

The classification included the number of feeding vessels into the bAVMs (<3 pedicles = 1 point, 3–6 pedicles = 2 points, more than 6 pedicles = 3 points), the eloquence of adjacent areas (non-eloquent = 0 points, eloquent = 1 points), and the presence of fistulous components (no = 0 points, yes = 1 point) (48). Puerto Rico grade ≤ 2 reliably predicted successful lesion obliteration with isolated endovascular therapy, whereas grades ≤ 3 were proposed strongly associated with cure after multimodality treatment and favorable neurological outcome. There was a stepwise increase in complications with the increase in Puerto Rico grade.

**Recommendations**

- In the judgment of the eloquent area, the eloquent cortex, subcortical fiber tracts, hippocampus, and the important cognitive brain area should be taken into consideration (*Class I; Level of Evidence B*).
- Pre- and post-interventional neurological assessment should be performed regarding the potentially injured neurological and cognitive functions. Muscle strength scale with KPS score or mRS score should be used for the motor evaluation; the Edinburgh Dominance Scale and language scales such as the West Aphasia Battery is recommended for the language evaluation; for patients with lesions involved in visual areas, vision and visual field examination is recommended. Mini-Mental State Examination (MMSE) and Montreal Cognitive Assessment (MoCA) are recommended to apply in the cognitive examination. National Institutes of Health Stroke Scale (NIHSS) is recommended for comprehensive assessments (*Class I; Level of Evidence B*).
- Besides traditional MRI, MRA, CTA, and DSA scanning, functional-MRI scanning, and DTI tractography are also useful in judging eloquent cortex and white matter fiber tracts (*Class I; Level of Evidence B*).
- In pre-surgical evaluating of the microsurgical treatment of bAVMs, in addition to the traditional SM Grading Scale and Lawton-Young Scale, the HDVL system is helpful for post-surgical neurological outcomes evaluation (*Class I; Level of Evidence B*).
- The involvement of eloquent fiber tracts should be considered in the preoperative evaluation to improve its predictive accuracy (*Class I; Level of Evidence B*).
- In evaluating the outcomes of radiosurgical and endovascular treatment for bAVMs, the modified-RBAS and Puerto Rico scale are helpful for radiosurgical and endovascular treatment, respectively (*Class I; Level of Evidence B*).

### Treatment Modalities

Quality of life (QoL) after the treatments has been emphasized in recent years. The incidence of neurological deficits is regarded as a critical index in assessing the safety and efficiency of treatment. Thus, more attention had been paid to developing interventional techniques for neurological protection, and predictive tools for therapeutic risks evaluation (32, 40, 41, 44, 46, 49–55). Treatments of eloquent bAVMs have to achieve two goals: (1) the complete obliteration of nidus and arteriovenous shunt; and (2) the protection of neurological functions, which might severely impact postoperative QoL.

Treatments of eloquent bAVMs are carried by three elementary surgical methods, including microsurgical resection, endovascular embolization, and stereotactic radiosurgery (SRS). Microsurgical resection can be performed initially or subsequently to other treatments. Complete obliteration may be achieved in most cases receiving resection. Endovascular embolization is usually performed as a precursor to microsurgery and radiosurgery. Complete endovascular obliteration is seldom reported to be achieved in a single-staged or multi-staged therapy (56). Stereotactic radiosurgery is applied to the bAVMs in deep locations or small sizes as a primary or complementary treatment. In the treatment of complex eloquent bAVMs, different therapeutic elements are usually cooperatively utilized (AKA multimodality treatments). Reviewing current literature, indications of each therapeutic modality were concluded.

#### Microsurgery

Microsurgical resection is the most common approach to achieve the complete obliteration of bAVMs. A systematic review by van Beijnum et al. (56) reported that microsurgery achieved the highest complete obliteration rate (≈96%), comparing with that of endovascular embolization (≈13%), and stereotactic radiosurgery (≈38%). The complete obliteration of bAVMs can eliminate the morbidities and mortalities induced by its hemorrhage in the future.

##### Threshold of Microsurgery

Microsurgical resection is not recommended for all eloquent bAVMs. The neurological risk of microsurgical resection should be evaluated. As mentioned above, the SM grading system and its supplemented scale are useful in the evaluation of operative risks and the prediction of neurological outcomes (41, 43, 51, 57, 58). Five grades of the SM grading system are divided into three levels, low grade (grade I–II), medium grade (grade III), and high grade (grade IV–V) (3). Studies on low-grade bAVMs reported morbidities of neurological deficits to range from 0 to 6.6% after microsurgical resections (57, 59–66). The morbidity rate of bAVMs in SM grade II increased to 0–69.2% (65, 67, 68). However, the worst neurological outcome was not induced by the involvement of eloquence. The microsurgery on eloquent bAVMs in SM grade II resulted in a morbidity rate ranging from 0 to 9.5%. There were two subtypes of eloquent bAVMs in grade III, the subtype of S1E1V1 and S2E1V0. The morbidity of neurological deficits was reported to be 4.8–16.7% and 15–25%, respectively (66, 69–71). They were in similar rates with the subtype of S2E0V1, but much lower than that of S3E0V0 (70). In the eloquent bAVMs in SM grade IV and higher, neurological risks increased rapidly to as high as 38% in the mono-therapy of microsurgery (40, 51, 57, 72, 73). However, preoperative annual hemorrhagic rates ranged from 1.5 to 10.4% in lesions of SM grade IV and V (72, 74, 75), which suggested the need for microsurgery. Several case reports and series had reported the successful utilization of individualized multimodality treatment to cure high-grade bAVMs with satisfactory morbidity and mortality (76–85). Multimodality treatments refer to the combined therapies of more than one elementary surgical treatment. Microsurgery is involved in most combinations. The performing of multimodality treatment is primarily for tentative or salvage purposes for high-grade bAVMs. Further discussed referred to section Multimodality Treatment.

Lawton et al. (41) proposed the supplementary scale of the SM grading system to refine the prediction of neurological outcomes. The full scale (SM grading system + supplementary scale) had been validated in bAVMs with deep and superficial locations. According to the studies with the full scale applied, monotherapy of microsurgery could result in satisfactory neurological outcomes in eloquent bAVMs in grade V and the lower (58, 86, 87). For the lesions in higher grades, not enough data support the exclusive utilization of microsurgery.

Results of functional neuro-images help to predict the individualized neurological outcome of each eloquent bAVMs. Lin et al. (88) proposed the minimum lesion-to corticospinal tract distance (LCD) to be 5 ml to secure the motor functions. Afterward, Jiao et al. (32) proposed the HDVL scale, enrolling LED, and achieved higher predictive accuracy than the full scale. The worsening of neurological outcome occurred in 0% of lesions in HDVL grade I and II, 11.8% in grade III, 31.5% and more in grade IV–VI. Monotherapy of microsurgery was recommended in lesions < HDVL grade IV.

The brainstem is a critical location with dense fiber tracts and nervous nuclei. Different procedures and techniques had been piroposed to cure brainstem bAMVs (89–91). However, current studies only demonstrated the therapeutic outcomes of highly selected patients. Thus, microsurgery of bAVMs in critical locations is not recommended, unless the relevant progressive neurological deterioration or hematoma occupation could not be postponed by endovascular surgery or radiosurgery (92).

**Recommendations**

- The Spetzler-Martin Grading system and its supplementary grading system are recommended to be utilized primarily to evaluate the risk of microsurgical resection (*Class I; Level of Evidence B*). The HDVL grading system is recommended for patients who have received DTI and fMRI assessments (*Class I; Level of Evidence B*). Microsurgical resection is reasonable to perform on lesions under Spetzler-Martin grade IV, or the combined grade VI (Spetzler-Martin grade plus the supplementary grade), or HDVL grade IV (*Class IIa; Level of Evidence B*). Individualized multimodality treatments can be useful to the bAVMs with Spetzler-Martin grade ≥IV, or combined grade ≥VI, or HDVL grade ≥IV (*Class IIa; Level of Evidence C*).
- The microsurgical resection may be considered in bAVMs located in critical locations (brainstem, pons, medulla, mesencephalon, etc.) when bAVM-related neurological deficits or mass effect of hematoma are progressive, and cannot be postponed by endovascular or radiosurgical treatments (*Class IIb; Level of Evidence C*).

##### Elective, Semi-elective, and Emergency Microsurgery

For eloquent bAVMs that require microsurgery, timing of treatment differs across situations. The emergency operation is performed in urgent situations, such as the occurrence of life-threatening hematoma, without any delay to prevent death or serious disabilities. The semi-elective operation is performed on the patients with prior hemorrhagic presentation, progressive neurological deficits, or AEDs-resistant epilepsy to prevent deterioration or death. The semi-elective operation should be done as early as possible, but can be postponed for the thorough preoperative preparation and evaluation. The elective operation is performed to the patients without life-threatening risk, and carried out at the request of the patient, and availability of the surgeon and facility. Both elective operations and semi-elective operations are aiming at preventing the onset of (re-)hemorrhage or symptoms in the future to promote QoLs. To ensure optimal neurological outcomes, the criteria in the last section should be obeyed.

Intracranial hemorrhage was the major threat of bAVMs. It occurs in an annual risk ranging from 1.3 to 4.1% (93–97). For the bAVMs with rupture history, the annual hemorrhagic risk increases to 4.5–4.8% (94, 95), and as high as 6–15.8% in the first year (98–102). Angio-architectural Features had been reported to be associate with ruptures, including (1) aneurysms located in nidus, arterial feeders, or irrelevant arteries; (2) venous drainage anomaly, such as the stenosis, occlusion, ectasia, kinking, or reflex of draining veins, and occlusion of the sinus; (3) single arterial feeder with high blood flow (94, 103–106). Microsurgical resection could achieve complete obliterates in most cases (96% approximately), and significantly reduce the re-bleeding rate (107). Considering the high risk of rebleeding in the first year, microsurgery is recommended to perform, but can be postponed for a short time for preoperative preparations and assessments.

Another common presentation of bAVMs is seizure, occurring in ~17–30% of patients (108). The onset of seizure was suggested to be associated with hemosiderin deposition, mass effect with cortical irritation, hemodynamic modifications, and/or vascular remodeling leading to stealing, ischemia, and neuronal damage (109). The seizures could be progressive and impact QoLs. Josephson et al. (110) reported that 76% of seizures would develop into epilepsy. Microsurgical resection was reported to be effective in the control of seizures in bAVM patients with refractory epilepsy (111, 112). The semi-elective microsurgery is recommended when the onset of seizure had progressed. Other presentations of bAVMs consist of neurological deficits due to the steal phenomenon, and headache. In these hemodynamic related symptoms, microsurgical resection had been reported to be effective for their relief (113–117). For asymptomatic bAVMs, elective microsurgical resection is considered to prevent the occurrence of symptoms and suboptimal events mentioned above.

In patients aged ≤ 40 years, 33% of intracerebral hemorrhages were caused by the rupture of bAVMs (118). In the acute phase of hemorrhage, clinical outcomes were associated with both the grade of bAVM and the degree of subarachnoidal hemorrhage (119). Kuhmonen et al. (120) reported that an early extirpation of bAVMs and evacuation of massive hematoma resulted in optimal outcomes in over 55% of patients. However, before evacuating hematoma or resecting bAVM, two points should be clear. Firstly, the etiology of a hematoma should be identified, such as bAVM rupture, hypertension, or amyloid (4). Secondly, if the hematoma is induced by bAVM rupture, the morphology and angioarchitecture of the lesion should be ascertained comprehensively. CTA or DSA had been proven effective on the etiological diagnosis of hematoma (121–124). However, the routine workflow usually takes a long door-to-operation (DTO) time, which may be intolerant to patients with life-threatening hematoma or progressive neurological deterioration. The hybrid-modality treatment integrates endovascular intervention (including catheter angiography) with microsurgical operations in one operating room, which could effectively shorten the DTO time of emergency patients. Hybrid-modality treatment had been proven to be feasible in the emergency disposal of severe trauma (brain trauma included), complex thoracoabdominal aortic pathology (125–127). Under a definitive diagnosis, emergency evacuations of hematoma and control of acute bleeding is warranted in the event of life-threatening mass effect, regardless of whether it is associated with the bAVM. Superficial bAVMs in small size (≤ 3 cm) can be resected simultaneously in an emergency operation. Meanwhile, bAVMs in deep locations or with large sizes are recommended to be resected *via* semi-elective microsurgical operations.

**Recommendations**

- The semi-elective operation is reasonable for patients with any of the prior hemorrhagic presentations, progressive neurological deficits, or AEDs-resistant epilepsy (*Class IIa, Level of Evidence B*). A semi-elective operation is reasonable for bAVMs with angioarchitectural features, which imply high rupture risks (*Class IIa, Level of Evidence B*). Elective operation is probably recommended for patients without any features above (*Class IIa, Level of Evidence B*).
- In an emergency operation, the evacuation of hematoma and control of acute bleeding can be beneficial to the event of life-threatening mass effect, regardless of whether it is associated with the bAVM (*Class IIa, Level of Evidence B*). Simultaneous resection of superficial bAVMs in small size (≤ 3 cm) in the emergency operation is reasonable (*Class IIa, Level of Evidence C*). The semi-elective microsurgical operation is probably recommended for the deep located or large-sized bAVMs (*Class IIa; Level of Evidence C*).
- Hybrid-modality treatment is probably recommended for the diagnosis and subsequent treatment of every emergency intracranial hemorrhage (*Class IIa; Level of Evidence C*).

##### Strategy of Microsurgery

**Single Microsurgery**

The single microsurgical resection of eloquent bAVMs shares the same procedural process with the non-eloquent: (1) performing the craniotomy to expose bAVM and relevant vessels; (2) isolating and controlling the arterial feeders; (3) dissecting the nidus along edges from adjacent parenchymal and vascular structures; (4) coagulating and dissecting the draining veins; and (5) closing and suturing up the incision. In the microsurgery of eloquent bAVMs, steps from 2 to 4 are nuanced with the general ones, because of the adjacent or overlapping relation between nidus and eloquent structures. Approaches to eloquent bAVMs in deep locations, such as the insula, basal ganglia, thalamus, and callosum, should be planned precisely to protect the surrounding eloquent cortex and subcortical parenchyma (86, 128, 129). The circumferential dissection of the nidus should be limited as well, to keep the eloquence-related structures intact. Steiger et al. (90) reported their procedure to limit the damage to parenchyma around in a small group of cases, which was proposed to coagulate from draining veins to the nidus. Other microsurgical or endovascular techniques should be considered for the protection of eloquent structures.

##### Microsurgery in Multimodality Treatments

Multimodality treatments refer to the performing of different elementary surgical therapies in a single stage or multiple ones. In staged-multimodality treatments, the microsurgical resection is commonly performed as a conclusive treatment, or as an initial treatment in a few cases as well (see section **Detection** and **Treatment of Residue**). As a conclusive treatment, the microsurgical resection is utilized as a salvage treatment to residual bAVMs, that has not been completely obliterated by other treatments (78, 130). Meanwhile, the microsurgical resection can be performed systematically in some bAVMs, which have been down-graded by endovascular embolization or/and SRS from higher grades (76, 131–133). Studies on staged-multimodality treatments suggested that the prior endovascular or/and radiosurgical treatments could improve the microsurgical condition with lower risks and difficulties. However, it should be noted that the risks of prior treatments needed to be acknowledged. The most commonly utilized paradigms are microsurgical resection combining with prior endovascular embolization. The prior embolization is supposed to decrease the operative risks of subsequent microsurgery in the following aspects: (1) aiding the elimination of feeders from deep sites of the operative field (for example, perforating arteries and branches of posterior cerebral arteries), and (2) making the nidus dissection easier with clearer borders, which is important to diffusive nidus adjacent to eloquence (79, 133, 134). Besides, it has been proposed for neurological protection. Han et al. (91) and Wang et al. (84) reported their experience of applying the staged or hybrid paradigms to protect the neurological function of bAVMs in brainstem and eloquence, respectively. In their procedures, the nidus adjacent to eloquence had been embolized through the prior endovascular manipulations. The subsequent microsurgery only removed the nidus distal to the eloquence, while leaving the embolized nidus *in-situ*. Another paradigm of microsurgical resection combining with prior SRS was reported in a few cases. The prior SRS decreases the risk of microsurgery as well, by a different pathological process from endovascular embolization (76, 135). It usually requires probation of 3–5 years to evaluate the effect of the prior SRS before microsurgery (136, 137). However, the risks of prior treatment would not be diminished by the cooperation of multimodality treatments. Prior embolization and SRS were reported to share a similar rate of adverse events, morbidity, and mortality with their independent implementations (76, 79, 82, 132, 138). Most of the suboptimal events occur in the process of endovascular procedures, or the latency period before microsurgery.

Hybrid-modality treatment is the up-to-date paradigm, which combines endovascular and microsurgical procedures in a single stage without any interval (81, 139). It has been proved to be feasible in treating bAVMs, especially the eloquent ones (80, 84). The hybrid-modality treatment possessed the advantages of endovascular and microsurgical manipulations, and expanded the operative techniques. Wang et al. (140) reported their intraoperative transvenous embolization technique to patients with difficult arterial and venous approaches. Most importantly, latent risks in the intervals of staged treatments were fundamentally diminished in the hybrid-modality treatment. Brown et al. (141) reported their experience in 19 patients who received hybrid-modality treatment, in which neurological outcomes were similar with staged multimodality treatment without the occurrence of intracranial hemorrhage after embolization or microsurgery. Hybrid-modality treatment may be a safer method of curing eloquent bAVMs radically and effectively, but need to be further validated.

**Recommendations**

- Multimodality treatments with microsurgery involved are probably recommended for the treatment of eloquent bAVMs in high grades. Downgrading the lesion before microsurgery can be useful to decrease operative risks and protect neurological functions (*Class IIa; Level of Evidence B*).
- The staged multimodality treatment of the microsurgery subsequent to endovascular embolization can be useful for treating bAVMs with diffusive nidus or feeders from deep sites of the operation field, which may reduce the risks of intraoperative bleeding and neurological deficits of the subsequent microsurgery (*Class IIa; Level of Evidence B*).
- Hybrid-modality treatment is probably recommended with similar safety and efficiency to the staged multimodality treatment, but with fewer risks than the latter (*Class IIa; Level of Evidence C*).

##### Detection and Treatment of Residue

Although microsurgical resection has achieved the highest complete obliteration rate among treatments, residual nidus can still be detected in follow-up angiographies. The cumulative incidence of residuals and recurrences had been reported to be 3% for SM grade I-II and 8% for SM grade III or higher (142, 143). The cause of residue is induced by the absence of adjunctive tools for an angiogram or bAVM detection. Most residues can be quickly detected through intraoperative DSA (80, 139, 144). Meanwhile, omissions might occur in intraoperative DSA. Aboukais et al. (145) reported that intraoperative or early postoperative angiography did not ensure the cure of bAVMs in several pediatric cases. Residues may be caused by vasospasm or recanalization of abnormal vessels. Delayed angiographies, *via* DSA, CTA, or MRA, were suggested in follow-ups, especially to pediatric patients (see section **Follow-Up**). It should be noticed that residual dysplastic vessels after cerebral arteriovenous malformation resection might be confounded with residual nidus (146). Residual dysplastic feeding vessels without an early draining vein do not necessarily represent residue after resection.

Intraoperative Doppler ultrasound can be used to detect the residual nidus. Four parameters could be obtained, including peak systolic velocity (PSV), end-diastolic velocity (EDV), mean flow velocity (MV), and resistance index (RI). RI (defined as the ratio of PSV to EDV) is regarded as the key parameter to identify the residual nidus. It has been generally accepted that RI is between 0.55 and 0.75 for the normal internal carotid artery, and higher in branches downstream (147). Arterial vessels with an RI lower than 0.55 should be noticed. Griffith et al. (148) reported their RI range of arterial feeders to be 0.14–0.50. With the enhancement of contrast, ultrasonographic angiography is able to help identify arterial feeders both on the surface and deep in the tissue (149, 150). Doppler ultrasonography cannot replace the intra/post-operative DSA. Regions of low-velocity blood flow or areas with very small vessels (i.e., much smaller than 0.6 mm in diameter) might be missed by ultrasonography. Hence, abnormalities such as venous angiomas on cryptic AVMs with low-velocity blood flow or thrombosed vessels may not be discernible in sonography. So the intraoperative ultrasound findings should be confirmed by angiography (147).

Patients with residual and recurrent nidus after microsurgery are still exposed to the threats of hemorrhage throughout their lives (135, 151). Only a few case-reports demonstrated the spontaneous thrombosis occurring in residual nidus (152, 153). The expectant or conservative treatment of residual bAVMs has not been widely accepted by neurovascular surgeons (152). The residue of bAVMs is usually disposed of through microsurgery, endovascular embolization, and SRS. A salvage microsurgical resection could be performed in one session with the assistance of intraoperative DSA (144, 154, 155), or another operation (146). Neurological risks of salvage microsurgery should be considered. SRS and endovascular embolization were proposed to be utilized for the salvage treatment of residues adjacent to eloquence or in a deep location (128, 156–158). Indications of endovascular embolization and SRS should be obeyed, respectively.

**Recommendations**

- Intraoperative or early postoperative DSA is recommended for detecting residual nidus (*Class I; Level of Evidence B*).
- Intraoperative Doppler ultrasonography is useful to primarily detect the residual nidus (*Class I; Level of Evidence B*). The complete obliteration should still be confirmed by a subsequent DSA.
- Repeat angiographies in follow-ups within 3–5 years after surgical resection is reasonable for detecting missed residues or recurrence (see section **Follow-Up**) (*Class IIa; Level of Evidence B*).
- Residual nidus after microsurgery is still exposed to the risk of hemorrhage. Salvage surgical treatments are recommended for the disposition of residues, including microsurgery, endovascular embolization, and SRS (*Class I; Level of Evidence B*). Subsequent SRS and endovascular embolization can be effective in achieving complete obliterations in residues adjacent to eloquence, following their indications, respectively (*Class IIa; Level of Evidence C*).

##### Surgical Adjuncts

Surgical adjuncts are indispensable for intraoperative localization and mapping of eloquent (sub-)cortical structures, including neuro-navigation, electrical cortical stimulation with awake craniotomy, and transcranial magnetic stimulation.

Most neuro-navigations are utilized based on structural and functional MRI. Data reconstructions should demonstrate the 3-dimensional information of brain, lesion, and eloquent structures. Despite providing a wealth of information, neuro-navigation was suggested to be ineffective in improving the neurological outcomes of microsurgeries in a randomized controlled trial (159). This was attributed to the different resecting strategies of bAVMs and brain tumors. The resections of a tumor could be stopped when reaching the edge of eloquence. By contrast, the resection of bAVMs cannot be stopped until completely removing the nidus. The alert of neuro-navigation hardly affects the damage to eloquence. However, neuro-navigation is not useless in the microsurgery of bAVMs. Torne et al. (160) reviewed the bAVMs surgically treated by Michael Lawton and emphasized the importance of identifying the location and border of the nidus, which could reduce the rupture risk during operation and improve clinical outcomes. Although neuro-navigation was proved to be ineffective in improving neurological outcomes, it was helpful to reduce operative risks by clearly demonstrating the localization and borders (88, 161–163). Intraoperative three-dimensional (3D) ultrasound was usually used as an assistance to the neuro-navigation to correct the brain-shift induced by craniotomy (164–168).

Intraoperative direct electrical stimulation (DES) on the cortex is regarded as the gold standard of eloquent mapping (169). It allows a safe real-time identification and hence preservation of essential pathways for motricity, sensibility, language, and even memory in the treatment of brain tumor and cerebrovascular diseases under general or local anesthesia (awake craniotomy) (170–172). Currently, the mapping through DES was usually performed on basis of fMRI (173, 174). Besides the direct stimulations to the cortex, Gamble et al. (175) revealed the value of subcortical stimulation on identifying subcortical eloquent structures. Concluding the proposals of current studies, the electrical cortical/subcortical stimulation was recommended if the lesion (1) adjacent to eloquence on fMRI (not distancing nor overlaying); (2) in large sizes and high SM grades; (3) with diffusive nidus. Differences existed in the application of DES between general and local anesthesia. The DES under local anesthesia had become popular in recent years. It was capable of mapping not only essential cortico-subcortical areas of motricity, but also areas of sensitivity, language, and even memory (174, 175). It had been proved to be effective in helping identifying eloquence and preserving neural functions (171, 176). Although DES under local anesthesia could make awake patients without any pain or discomfort (177, 178), the psychological effect still needed concern. By contrast, the DES under general anesthesia was performed earlier, usually accompanying with electrocorticography (ECoG) and neurophysiological monitor. Its capability of eloquent mapping was limited to motor and somatosensory areas, in which optimal neurological outcomes could be achieved (170, 171). The ECoG, which used accompanying DES, was proved to be effective in identifying epileptogenic cortex for subsequent surgical management (179, 180). Despite its high accuracy and efficiency, DES faces a similar situation with fMRI-based neuro-navigation. Mapping of the eloquent area could take effect as an alarm to the neurosurgeon, but helpless to limit the extensive excision. Besides, DES might cause generalized seizures and result in disastrous consequences (171). The utilization of DES remains controversial and needs further investigation. The requirement of gross identifying of motricity and somatosensory area can be met by a neurophysiological monitor.

Navigated transcranial magnetic stimulation (nTMS) is applied to the preoperative mapping of cortical motor and language regions in recent years. The comparison between nTMS and DES resulted in similar accuracies (181–185). Besides, nTMS had been proved to be superior in mapping the eloquence to other non-invasive techniques, such as fMRI and magneto-encephalography (185–187). Ille et al. (188) reported their experience of utilizing nTMS to fix the mapping result of fMRI. Kronenburg et al. (189) reported the utilization of TMS to non-cooperative patients and achieved satisfactory results. Although nTMS is widely applied in brain tumors, it has seldom been used for cerebrovascular disease. The preparation and implementation of microsurgery are different between tumors and bAVMs. More studies are needed to validate its safe utilization to bAVMs, especially on seizure and hemorrhage-related complications.

**Recommendations**

- The utilization of fMRI-guided neuro-navigation does not improve neurological outcomes, but can be useful for locating and delineating the lesions (*Class IIa; Level of Evidence B*). Intraoperative 3D ultrasound is effective in correcting the brain-shift induced by craniotomy (*Class I; Level of Evidence B*).
- Intraoperative direct electrical stimulation (DES) is feasible on mapping motricity eloquence under general, and other sensibilities, language, and even memory eloquence under general anesthesia. DES might be considered for delineating the nidus adjunct to eloquence, or in large sizes, or with diffusive nidus. The risks and psychological impacts of DES are not well-established (*Class IIb; Level of Evidence C*).
- Neurophysiological monitor can be useful for gross mapping of motricity and somatosensory areas (*Class IIa; Level of Evidence C*).
- Electrocorticography (ECoG) is recommended for localizing epileptogenic cortex (*Class I; Level of Evidence B*).
- Navigated transcranial magnetic stimulation (nTMS) has been utilized for mapping and fixing mapping results of fMRI in the preoperative preparation, mostly in brain tumors and seldom in bAVMs. The risks of nTMS are not well-established (*Class IIb; Level of Evidence C*).

#### Endovascular Neurosurgery

Endovascular embolization (EE) occludes blood flow by delivering occlusive agents into the feeding arteries and nidus through microcatheters (1, 190). The role of endovascular embolization in treating bAVMs includes (1) as a therapeutic strategy; (2) as palliative or targeted strategies; and (3) as adjuvant management before microsurgical resection or SRS, to minimize the risk of hemorrhage (191, 192).

##### Curative Embolization

Monotherapy of curative embolization was believed to be difficult to completely eliminate bAVMs, with rates of angiographic obliteration ranging from 13 to 96% (56, 193–195). Complete cure was attainable for small-sized (≤ 3 cm), superficially located bAVMs (194). Angioarchitectural characteristics with a single feeding artery also achieved favorable outcomes (196). Properly applied EE might decrease the size and grade of bAVMs without sudden changes of pressure and reduce the risk of adjacent arterial recruitment (106). The overall complication rate of endovascular therapy for bAVMs was 25.0%, with an incidence of 6.6% in permanent neurological deficits (56). The number and diameter of feeding arteries, nidal volume, deep venous drainage, and eloquent location were risk factors of embolization-related complications (47, 54, 55). When disposing of bAVMs in corpus callosum with complex angioarchitectures and eloquence involved, curative embolization achieved complete obliteration in only 40–60% of cases and hemorrhage complications occurred in 7% of cases (197, 198).

Strategies of embolization have been discussed. Sahlein et al. (199) proposed that the single-stage embolization reached a lower rate of mortality and morbidity than the multi-staged embolization. It had been demonstrated that staged embolization was an independent risk factor for unfavorable outcomes after embolization (200, 201). It was supposed to attribute to the recanalization and recruitment of arterial feeders during staged procedures (199). Even so, the staged embolization had been used as a strategy to reduce the risk of normal perfusion pressure breakthrough by progressively minimizing the blood flow, particularly for medium-large bAVMs (>3 cm) (202, 203). Previous studies had supported that the interval between each session should be 4–6 months when applying the staged embolotherapy (202, 203). Ma et al. (204) reported that staged embolization was effective in treating eloquent bAVMs with large sizes. Different paradigms of staged embolization were proposed without a consensus. Ma et al. (204) reported their paradigm, which achieved an obliteration rate within 60% in the initial session, and achieved complete occlusion in 2–3 months through the following 1–2 sessions. Katsaridis et al. (202) reported another paradigm, which proposes to embolize ≤ 30% of nidus in each session. The optimal paradigm of endovascular embolization remains to be researched.

**Recommendation**

- Curative embolization of small-sized (≤ 3 cm), superficially located bAVMs with a single feeding artery can be useful (*Class IIa; Level of Evidence B*).
- Staged embolization can be beneficial for curing medium-large bAVMs (>3 cm) by gradually reducing the risk of NPPB, but the probability of recanalization remains (*Class IIa; Level of Evidence B*).
- The interval between each stage and extent of embolization has not been well-determined (*Class IIb; Level of Evidence C*).

##### Palliative Embolization

The selective embolization of high-flow feeding arteries might postpone the progression of frequent seizures or neurological deficits caused by venous hypertension and arterial steal syndrome (106). A retrospective study suggested that the partial embolization might result in complete occlusion and improve survival rates, comparing with conservative treatment in the long-term (205). Flores et al. (206) proposed to utilized the palliative embolization to symptomatic bAVMs in Spetzler-Martin grade IV or V, that are inadvisable for surgical resection or SRS. Although the palliative embolization might improve the clinical manifestation of patients by changing the hemodynamics of lesion (207, 208), it was accused of increasing the risk of hemorrhage in large bAVMs (75). The role of palliative embolization remains controversial.

**Recommendation**

- Palliative embolization remains controversial. The selective embolization of high-flow feeding arteries might be considered to postpone the progression of seizures or neurological deficits in surgical/SRS-inadvisable bAVMs, while potentially increasing the risk of hemorrhage (*Class IIb; Level of Evidence B*).

##### Targeted Embolization to “Weak Point”

Targeted embolization aims at eliminating the “weak point” of bAVMs, including intranidal or flow-related aneurysms, high-flow arteriovenous fistulas, and venous flow obstruction, and other anigoarchitectural features with high rupture risks (3, 101, 106, 209). Targeted embolization is proposed to perform when definitive managements were infeasible or excessively risky. Targeted embolization was reported to be performed to the ruptured bAVMs in the acute phase (210), and restoration stage (211). The effect of embolization in the management of ruptured bAVMs has not been well-established. The targeted embolization was supposed to reduce the incidence of bleeding after radiosurgery (106).

**Recommendation**

- Targeted embolization is recommended for bAVMs with a “weak point” when definitive treatments are infeasible or too risky (*Class I; Level of Evidence B*).

##### Pre-microsurgical Embolization

Pre-microsurgical embolization has been used as the most common adjunct to improve the therapeutic safety and efficiency of bAVMs (79, 80, 199, 212). The pre-microsurgical embolization resulted in permanent morbidity of 2.5% and a mortality of 2.0% (201, 213). The strategy was proposed to facilitate the subsequent microsurgery in the following aspects: (1) occluding the supply arteries and lowering the size of bAVMs, to minimize the risk of bleeding, (2) eliminating the deep perforators that are inaccessible for surgeries, (3) embolizing the flow-related aneurysms (56, 82, 195, 206, 214, 215).

The prior embolization before microsurgery was proposed to minimize the risk of NPPB by gradually reducing the blood flow before surgery, and normalize the hemodynamics for high-flow or large lesions (202, 216–218). Whereas, the vigilance of the potential risks (e.g., hemorrhage, infarction, or seizures) in-between the treatments should be considered as concerns (219, 220). The subsequent microsurgery could be performed consecutively after the embolization in one session, which was known as a hybrid-modality treatment (80). It made the manipulations of embolization more flexible, and overcame the disadvantages and limitations when solely performed. It had been shown that the staged paradigm increased the expenditure of treatments (200, 221). The hybrid-modality treatment might reduce the frequency and duration of anesthesia and operation by a single treatment, from a health-economics perspective. However, the optimal paradigm of preoperative embolization and subsequent microsurgery remained unclear (82). The previous series had performed a paradigm with an interval between each embolization therapy for 16–42 days, and an interval before surgery for 1–42 days (79, 212, 222, 223). Nataraj et al. (224) supported a prompt microsurgical resection after endovascular intervention for a lower rate of mortality and morbidity.

**Recommendation**

- The pre-microsurgical embolization is probably recommended for the bAVMs with large size, or deep perforating feeding arteries, or inaccessible locations for surgeries, or concomitant flow-related aneurysms (*Class IIa; Level of Evidence B*).

##### Pre-radiosurgical Embolization

The pre-radiosurgical embolization has been proposed to decrease large bAVMs to a suitable size for the subsequent SRS. It was particularly recommended for the lesions ≥3 cm in diameter, or the lesions with relevant aneurysms or high-flow fistulas. A pre-radiosurgical embolization could minimize the risk of hemorrhage before the definitive obliteration by SRS, especially effective for ruptured bAVMs in the posterior fossa (1, 3, 42, 225, 226). The multimodality treatment, consisting of prior embolization and subsequent SRS, was reported to achieve a complete obliteration rate of more than 60% in large bAVMs (227, 228). The efficiency of the subsequent SRS might be improved if the volume of the residual nidus is no more than 10 cm^3^ after embolization (77). The drawbacks induced by prior embolization needed to be considered. Embolic agents might shield the nidus from the radiation as protectants and make the outlines obscure with subsequent targeting inaccurately (229). Additionally, embolization might decrease the rate of obliteration by facilitating angiogenesis (230). The recanalization of embolized arteries might result in delayed recurrence (77). Due to the limitations, this paradigm might worsen the outcome of bAVMs (231–233).

Eloquent regions of basal ganglionic and thalamic AVMs could be treated with embolization in conjunction with SRS. Complete obliteration was observed in 14.3% and improved disabling in more than 1/3 of patients (234). Also, selected brainstem AVMs could be treated with embolization combined SRS, while the selection criteria had yet to be determined. Favorable outcomes were potentially comparable with general bAVMs under precision techniques (235).

**Recommendation**

- Pre-radiosurgical embolization may be considered for reducing the size of bAVMs, particularly for large lesions (>3 cm), or occluding bAVM-related aneurysms or high-flow fistulas, whereas the effectiveness remains uncertain (*Class IIb; Level of Evidence B*).

##### Seizure Control

Endovascular embolization had been reported to be less effective in controlling bAVM-induced seizures. Hyun et al. (236) investigated 399 bAVM patients with long-term follow-up after embolization, and found out that only half of the patients achieved seizure-free status after embolization, compared with 78 and 66% in the surgical and SRS groups, respectively. The duration of seizure-free status was 8.1 months in the embolization group and 20.5 months in the SRS group. A meta-analysis demonstrated that embolization resulted in the highest morbidity of new-onset seizures (39.4%), compared with other treatments (111).

**Recommendation**

- Embolization is not indicated for seizure control (*Class III; Level of Evidence A*).

##### Embolic Agents

Varieties of embolic agents are available in the endovascular treatment of bAVMs, consisting of adhesive, non-adhesive, and solid ones.

The adhesive embolic agents include N-butyl 2-cyanoacrylate (NBCA) and NBCA metacryloxysulfolane (NBCA-MS). Both NBCA and NBCA-MS have been proved to be efficient and safe in treating bAVMs with sophisticated endovascular skills (237–239). Compared with NBCA, the main advantage of NBCA-MS has a longer polymerization time (NBCA vs. NBCA-MS = 15–40 s vs. 60–90 s), which provides a sufficient and precious time window for its diffusion in bAVMs (240).

The non-adhesive embolic agents mainly include Ethylene vinyl alcohol (EVOH) and ethylene vinyl copolymer (EVAL). Comparing with NBCA, EVOH achieved indifferent results in its safety validations (241, 242), and was proved to be higher efficiency in occluding arterial pedicles (221). The comparative study on EVAL has not been widely conducted. Although the non-adhesive ones have better performance in endovascular embolization, both EVOH and EVAL have to be used accompany with dimethyl sulfoxide (DMSO), the latter of which might induce a series of side effects due to its vascular toxicity (243).

The success in embolization relies on the appropriate choice of embolic agent. the polymerization speed of NBCA could be reduced by adding lipiodol. NBCA is usually used in a concentration of 16–50%. Higher concentration results in higher polymerization speed. Commercially available EVOH is premixed in different concentrations, including 6 and 8%. Higher concentration results in higher polymerization speed. Choo et al. (244) reported their experience of using EVOH and NBCA in high concentration and coil to an embolize dural arteriovenous fistula. EVOH embolization was proved superior to NBCA and coil embolization in completely obliterating DAVFs.

**Recommendation**

- Non-adhesive embolic agents are probably recommended to the adhesive ones for the complete embolization of bAVMs (*Class IIa, Level of Evidence C*).
- For bAVMs with high flow capacity, a non-adhesive embolic agent in high concentration is probably recommended (*Class IIa, Level of Evidence C*).

#### Stereotactic Radiosurgery

Stereotactic radiosurgery (SRS) has been widely accepted as an effective treatment for patients with small bAVMs, especially for those with deep location or eloquence involved (44, 245, 246). SRS leads to proliferation of endothelial cells, progressive, concentric vessel wall thickening over years, and eventually endoluminal occlusion of the bAVM nidus (247). Obliteration of the bAVM is the goal for SRS. The disadvantage of SRS compared with microsurgery or embolization was the latency interval between treatment and obliteration, which differs from 6 months to several years (135, 248). Patients were remained at the risk of hemorrhage and delayed presentation of procedural complications during the latency interval. The annual risk of post-SRS hemorrhage was reported as 1.1% (249). Actuarial obliteration rates after SRS were related to multiple independent variables, and generally ranged from 27 to 62% within 3–10 years of treatment according to a multicenter retrospective cohort (248). The Spetzler-Martin grading scale was the most commonly used system for stratifying bAVMs, there were some grading scales used to predict SRS outcomes for bAVMs such as VRAS and RBAS (44, 46).

##### Small Size

Multiple studies were indicating that SRS appeared to be best suited for small volume bAVMs, which were <10 cm^3^ in volume or <3 cm in its maximum diameter (44, 231, 250–252). However, most of these studies were retrospective single-centered cohorts. Graffeo et al. (253) systematically reviewed eight studies with 1,102 bAVMs involved, and proposed that SRS appeared to be a safe, effective treatment for bAVMs in Spetzler-Martin grade II and might be considered a front-line treatment, particularly for lesions in deep or eloquent locations. A cohort study on SRS with 363 basal ganglia or thalamic bAVMs suggested its preference for the majority of basal ganglia and thalamic lesions. Another cohort study with 891 bAVMs (eloquence involved in 89.8%) in Spetzler-Martin grade III, suggested that the lesion with small sizes (maximum diameter <3 cm) had the best outcomes after single-staged SRS, even with critical structures involved (229).

**Recommendation**

- SRS can be effective in treating small-sized (≤ 3 cm) bAVMs in deep eloquent areas, including those located in the basal ganglia, thalamus, corpus callosum cerebellum, and brainstem (*Class IIa; Level of Evidence B*).

##### Medium and Large Size

The medium and large-sized bAVMs referred to those with a maximum diameter of 3–6 and ≥6 cm, respectively. Those lesions usually belonged to Spetzler-Martin grade III–V.

The traditional paradigm of single-session radiosurgery was not usually used for bAVMs larger than 3 cm in diameter, because of its low total obliteration rate (254). In a retrospective multi-centered study, 233 bAVMs in Spetzler-Martin grade IV (94.4%) and V (5.6%) were treated with single-session SRS (229). A limited role of single-session SRS was suggested in the management of high-grade (IV–V) bAVMs and particularly in the ruptured ones (255). The benefit of SRS for medium-size bAVMs in Spetzler-Martin grade III (i.e., 3 cm < those <6 cm in maximum diameter) was also less evident.

Meanwhile, the bAVMs in Spetzler-Martin grade IV–V were usually with larger volume, more complex angioarchitectures, and frequently located in critical locations. There was no consensus on the optimal management of these high-grade bAVMs, SRS was proposed to be one of the treatments that could be utilized (72, 255). Staged SRS was optional for large bAVMs, but usually utilized in multimodality treatments with mixed results (76). The therapeutic paradigm could be staged by the dosage of radiation and volume of the lesion. A dose-staged SRS treated the entire volume with a repeated low-dosage SRS; and the volume-staged SRS provided a sufficient therapeutic dosage to the targeted volumes as a part of the lesion (256). A systematic review suggested that the volume-staged SRS could achieve a higher obliteration rate and similar complication rate compared with the dose-staged one in the treatment of bAVMs in large volume (>10 cm^3^) (257).

**Recommendation**

- Single-session SRS is not recommended for patients with large-sized (>3 cm) bAVMs, especially for those which are ruptured (*Class III; Level of Evidence B*).
- Staged SRS might be considered for treating large bAVMs, however, the effectiveness of staged SRS is unclear (*Class IIb; Level of Evidence C*).
- Volume-staged SRS is probably recommended in preference to the dose-staged treatment (*Class IIa; Level of Evidence B*).

##### SRS After Endovascular Embolization

It had been reported that the prior embolization of bAVMs would lower the obliteration rates of SRS (231, 249, 255). The prior embolization was proposed to promote the angiogenesis of bAVMs, which might increase the radio-resistance of the lesion and decrease its obliteration rate (258). However, the multimodality treatment of SRS plus prior embolization had been proposed to benefit outcomes for high-grade bAVMs (259). The timing of SRS after embolization had not been determined, which could range from days to years (3 months in median) (225, 260). Referring to the stereotactic radiosurgery guideline for bAVMs, several weeks of latency after the prior embolization was considered beneficial to reduce the risk of post-radiosurgical complications (261).

**Recommendation**

- The SRS subsequently to endovascular embolization can be generally beneficial to high-grade bAVMs, though the obliteration rates are lower in embolized lesions (*Class IIa; Level of Evidence C*).

##### Associated Aneurysm

The presence of an untreated bAVM-associated aneurysm was proposed to be a strong predictor for post-SRS hemorrhage (229, 262). AVM-associated aneurysms should be obliterated *via* microsurgery or endovascular surgery to reduce the hemorrhage risk during the latency interval (248).

**Recommendation**

- It is recommended to treat bAVM-associated aneurysms before SRS to reduce the risk of post-SRS hemorrhage (*Class I; Level of Evidence B*).

#### Multimodality Treatment

Multimodality treatments of bAVMs included different combinations of mono-therapeutic elements, such as microsurgery, endovascular embolization, and SRS. Varieties of multimodality modes had been developed to reduce the postoperative morbidity and mortality of bAVMs. Most of them were utilized for the treatment of high-grade bAVMs, which were difficult to cure by any monotherapy, or to exceed the indications of it (76, 78, 83, 158, 263). It could be applied to bAVMs in low grades as well, for the specific purpose of protecting neurological functions and decreasing intraoperative risks (84, 132, 133). However, no extra benefit had been observed in the low-grade lesions (131). The therapeutic modes and strategies of multimodality treatments have been interpreted in relevant sections above.

**Recommendation**

- Multimodality treatments are reasonable for the treatment of high-grade bAVMs, which are difficult to cure by any monotherapy, or to exceed the indications of it (*Class IIa; Level of Evidence C*).

#### Conservative Treatment

Current conservative treatments cannot promote the obliteration of bAVMs, however, they were preferred reluctantly under a few certain conditions, especially for those located in critical locations. In a prospective study with 48 deep located bAVMs (in basal ganglia, thalamus, insula, etc.) the outcomes of 12-year follow-up indicated that conservative treatments resulted in better prognosis in unruptured bilateral thalamic bAVMs of Spetzler-Martin grade V (86). Another research by Potts et al. (223) also supported the conservative treatment to the unruptured thalamic bAVMs. For asymptomatic large (>6 cm) brain stem AVMs, Thines et al. (264) suggested that the surgical intervention would increase the risk of neurological deterioration by 16-fold at final follow-ups. Spetzler and Martin (51) supposed that the large diffusive bAVMs dispersing through critical areas were inappropriate for microsurgery alone.

Conservative treatments were proposed to be optimal in the management of unruptured eloquent bAVMs. ARUBA trial was the first randomized controlled trial focusing on these issues. Two-hundred and twenty-six adult patients (18 years or older) were recruited during 2007–2013 and randomly allocated to medical management alone (*n* = 110) or interventional therapy (*n* = 116) including resection, embolization, SRS, and multimodal approaches (97). The published results of ARUBA suggested that medical management resulted in lower risks of stroke or death in the 33 months of follow-up than interventional management (10.1 vs. 30.7%) in the patients with mRS ≤ 1, or bAVMs lower than Spetzler-Martin Grade IV (62% of ARUBA cases were in grade ≤ II), or bAVM sized <60 mm (62% of lesions in ARUBA sized <30 mm). It was halted because the interim results showed the superiority of the medical management group. It seemed that the conclusion of ARUBA strongly supported the conservative management to eloquent bAVMs, however, it was argued for its limitations. ARUBA trial was criticized for its low enrollment rate, insufficient sample size and follow-ups, high interventional hemorrhage rate, and lack of treatment stratification (265, 266). The study initially estimated that 800 patients would be selected based on statistical analysis. Due to the difficulty of enrollment, 223 cases were included in the analysis which affected the statistical results. The insufficient duration of follow-up might also omit the hemorrhagic risks in long term and over-amplified the short-term complications of the interventions (267). Studies of ARUBA-eligible patients had reported more favorable results and substantially less morbidity, compared to the outcomes of ARUBA (268–270). In the retrospective study of 142 ARUBA-eligible patients treated with embolization, surgery, and/or proton beam radiosurgery during 5 years of follow-up, the risk of stroke, death, and progressive symptoms are less in the intervention group. For those younger patients, conservative management may be inappropriate due to the high accumulative risk of hemorrhage, considering that the annual risk of hemorrhage may be as low as 1% or as high as 33%.

Anti-epilepsy drugs (AEDs) are the essential management for patients with bAVM-related epilepsies. Monotherapy of AEDs was taken by 57% of bAVM patients with epilepsy presentation. However, Josephson et al. (110) reported that AEDs had limited effect on reducing the seizure risk in patients with ruptured temporal bAVMs. If anticonvulsant therapy failed to control seizures, surgical management might be pursued (271).

No specific medicine has been applied for the treatment of bAVMs. Therapeutic strategies like anti-angiogenesis drugs, immunomodulatory drugs, and anti-inflammatory drugs which aim at preventing hemorrhage are still in the experimental stage (272). Headache occurred in ~5–14% of patients with bAVMs, and it could be unilateral or bilateral concurrent with migrainous features. No validated therapy has been applied to release the headache (273, 274).

**Recommendations**

- Conservative managements are reasonable for large-sized (>6 cm) unraptured bAVMs of adult patients concurrent with one of the following conditions: (1) bilateral thalamic bAVMs with deep venous drainage that are deemed inoperable, (2) asymptomatic patients with unruptured bAVMs involving brain stem parenchyma, or (3) diffusive bAVMs dispersed through eloquent areas (*Class IIa; Level of Evidence C*).
- The effectiveness of conservative management on ARUBA-eligible patients is uncertain, due to the limitations and disputed conclusions of the ARUBA trial (*Class IIb; Level of Evidence B*).
- The monotherapy of AEDs can be useful to control the bAVM-related epileptic seizure diagnosed by an electroencephalograph (*Class IIa; Level of Evidence C*). The usefulness of prophylactic use of AEDs is uncertain (*Class IIb; Level of Evidence C*).

### Follow-Up

Both neurological and neuroimaging evaluations should be involved in the follow-up of eloquent bAVMs.

Neurological evaluations should be subjectively and objectively conducted. Subjective neurological evaluations, such as the most commonly used modified Rankin Scale (mRS), Glasgow Outcome Scale (GOS), and Karnofsky Performance Scale (KPS), could reflect the QoL of patients. Meanwhile, Objective evaluations were necessary for directing subsequent treatments or rehabilitations, and for outcome assessment. Neurological physical examination (PE) should be performed in the face-to-face follow-ups. Different neurological functions should be specifically noticed for the lesions in different localizations, such as cognitive and orientating functions for frontal lesions, linguistic functions for left perisylvian fissure lesions, the visual field for occipital lesions, fine and gross motor functions for precentral gyrus and supplementary motor area, and coordinating and fine motor functions for cerebellar lesions. Results of neurological PE should be described in detail in medical records for dynamic evaluations. A reasonably accurate NIHSS could be reconstructed from a well-documented medical record for trial-usage (275).

Feasible neuroimaging evaluations include digital subtraction angiography (DSA), computed tomographic angiography (CTA), and magnetic resonance image (MRI) related scans (276–278). Neuroimaging evaluations were supposed to play critical roles in the detection of bAVMs, including their residue and recurrence (279). However, the optimal frequency and modality have not been well-defined.

The follow-up to untreated bAVMs aims at predicting their hemorrhagic risks by discovering risk factors. The frequency of follow-up for untreated bAVMs verifies with relevant factors, but had not been defined yet. Brain AVMs with the following features had higher (re-)hemorrhagic risks than the others: (1) primary hemorrhagic presentation, (2) in deep locations, such as insular, thalamus, basal ganglia, corpus callosum, brain stem, or cerebellum; or (3) exclusive deep venous drainage (95, 99, 103). The early identification of these features might influence the therapeutic strategy and prevent the potential hemorrhage. However, the impacts of gender, age, and nidal size remain controversial. In the bAVMs with a hemorrhagic presentation, the re-hemorrhagic risk changes along with time. Yamada et al. (99) proposed to perform follow-up every 3 or 6 months in their study and report the changes of risks. In the first year after the initial hemorrhage, the annual hemorrhagic risk was reported to be 15.42% for a subsequent hemorrhage. In the subsequent 4 years, the annual risk decreased to 5.32%. After more than 5 years, the annual risk further decreased to 1.72% per year. Meanwhile, the hemorrhagic risks remained unchanged at lower rates. The variation of (re-)hemorrhagic risks indicates different frequencies of follow-up. For the bAVMs without any hemorrhagic presentation, follow-ups should be performed annually with neuroimaging evaluations. For the patients with any risk factors of (re-)hemorrhage, neuroimaging follow-ups should be performed every 6 months. For the bAVMs which have ruptured within 1 year, neuroimaging follow-up should be considered every 3 months.

The follow-up to postoperative patients aims at timely detection of the residue or recurrence, and prevention of potential intracranial hemorrhage. The etiology of recurrence is not clear yet. Several mechanisms have been proposed to explain the pathological process of recurrence. The recurrence of bAVMs was mostly reported in studies on pediatric bAVMs (280, 281), and a few adult cases (138). The earliest recurrence of bAVMs had been detected in 3 months after the operation (282), and the latest in 17 years (276). Studies suggested DSA be more sensitive in detecting residue and recurrences (278). DSA was recommended at 1, 3, 5 years after treatment and every 5 years thereafter (283). By contrast, MRI might miss subtle bAVMs (277). MRI had been demonstrated to have 100% specificity, 80% sensitivity, and 91% negative predictive value for the identification of obliteration compared with angiography (284). Therefore, MRI was commonly used in the preliminary screening of recurrence followed by DSA performed on suggestive cases (281, 285–287), or in the patients who refused to receive DSA as an alternative. Computed tomographic angiography (CTA) is another minimally invasive method to detect postoperative recurrence or residue of bAVMs (283). The efficacy of CTA and MRA on detecting residual and recurrent bAVMs has been rarely compared. Giesel et al. (288) reported their results on 19 postoperative cases, which suggested that CTA was more sensitive in the detection of the residual bAVMs.

Functional MRI is seldom utilized in follow-up. A few descriptive studies reported the reorganization of language or motor cortex in adjacent or symmetric areas in postoperative patients (114, 289–291). However, the hypothesis of postoperative eloquent plasticity or reorganization remains controversial. The studies by Deng et al. (292, 293) revealed the existence of eloquence reorganization was before the intervention, which might weaken the meaning of fMRI follow-up. Besides, the methodological limitations of fMRI restrict the dependability of its results. The effect of fMRI in follow-up remains to be further investigated.

**Recommendations**

- Subjective neurological evaluations, such as mRS, GOS, and KPS, can be useful for the assessments of QoL (*Class IIa; Level of Evidence B*). Objective neurological evaluations by detailed neurological PE or NIHSS are recommended in face-to-face follow-ups, for specific evaluation of neurological outcomes (*Class I; Level of Evidence B*).
- Neuroimaging follow-ups are recommended for detecting bAVMs (residue and recurrence included) and preventing its rupture (*Class I; Level of Evidence A*).
- DSA is effective in detecting residue or recurrence and evaluating obliteration rates (*Class I; Level of Evidence B*).
- The angiographies of computed tomography and magnetic resonance can be useful as a preliminary screening method with following DSA to suggestive cases, or as an alternative to the patients refusing DSA (*Class IIa; Level of Evidence B*). It is reasonable to choose CTA over MRA in patients tolerant to X-rays (*Class IIa; Level of Evidence C*).
- For untreated bAVMs (conservative treatment included), neuroimaging follow-up is recommended annually for those without any hemorrhagic risk factors, in every 6 months for those with any (re-)hemorrhagic risk factors, and in every 3 months for those ruptured within 1 year (*Class I; Level of Evidence C*).
- For the postoperative patients, neuroimaging follow-ups should be performed as early as 3 months after treatment, and are recommended to be performed at 1, 3, 5 years after treatment and every 5 years thereafter (*Class I; Level of Evidence B*).

### Rehabilitation

Knowledge of the natural history of recovery pattern and prognosis for residual disability and functioning are limited. No specific rehabilitation strategy has been proposed to recover the neural deficits induced by eloquent bAVMs or the operation on them. Similar rehabilitation services are being performed on patients with neural deficits induced by intracranial hemorrhage, ischemic stroke, and operation. Specific rehabilitation strategies remain to be studied.

### Future Considerations

At present, neuroscientists have strived to investigate the comprehensive human brain network at the micro-, meso-, and macro-scale. Brain functional atlas based on resting-state magnetic resonance imaging (rs-fMRI) and task functional magnetic resonance imaging together with brain structural atlas would play a significant role in the understanding of brain functional connectivity and its dynamic behavior (294, 295). Meanwhile, with the development of the brain mapping technologies such as functional MRI, electrocorticogram (ECoG), transcranial magnetic stimulation (TMS), and positron emission tomography (PET), more brain functional areas and important brain network nodes or hubs would be recognized (296). The future brain functional protection would be developed toward the protection of more elaboratively neurological and cognitive function. Moreover, the development of technologies of brain-computer interfaces, such as the neural dust, and the study of the neural stem cells shed light on the neural rehabilitation of patients suffering from postoperative neurological deficits (297, 298).

Minimally invasive and non-invasive is proposed to be the development direction of eloquent bAVMs.

The standardized paradigms of endovascular embolization are urgent to direct clinical practice of endovascular surgeries, especially for the palliative and pre-radiosurgical embolization. Defects of endovascular material still limit the effect of embolization, including the poor controllability of the embolic agent and maneuverability of instruments, which induce a low rate of complete obliteration. The development of both endovascular materials [e.g., coils, balloons, polyvinyl alcohol particles, and n-Butyl Cyanoacrylate (n-BCA), and Onyx] and techniques (e.g., pressure cooker technique, dual-lumen balloon catheter technique) would promote its therapeutic effect to the final goal of complete cure (194, 299–301).

Optimization of radiosurgical planning is important to improve the total obliteration rate while maintaining reasonable safety. For example, A recent study proposed that in addition to keeping a minimal margin dose of 17 Gy, increasing the percentage of the bAVM volume that receives at least 20 Gy treated in two stages could improve the outcome for large-volume bAVMs (302). What is more, a novel deep learning-based method to automatically segment the bAVM volume may be helpful for radiosurgical planning (303). Further study for improving the treatment planning system of SRS is required.

The indication of surgical treatments is critically concerning, which has been simplified to ruptured and unruptured since the publishing of the ARUBA trial and its controversial results. For ruptured bAVMs, surgical treatments have never been disputed. The surgical management of ruptured bAVMs obeys the indications and contraindications proposed over the past few decades. A thorough investigation of literature ensures the applicability of recommendations in these guidelines to the ruptured bAVMs. For unruptured bAVMs, conservative treatment was proposed to result in significantly lower risks of death or stroke and better outcomes than surgical treatments (97, 304). ARUBA is the first randomized trial of unruptured bAVMs to better understand their natural history and associated treatment risks, however, it is controversial for its results. The limitations of its methodological design, trial implementation, and data interpretation were widely questioned (270, 305, 306). Studies revealed better or non-inferior results on morbidity and mortality in ARUBA-eligible patients who received microsurgical, endovascular, radiosurgical, and multimodality treatment, which is opposite to the results of ARUBA (266, 269, 307, 308). Thus, surgical treatments are feasible to unruptured bAVMs. Given the controversy, the indications of surgical management on unruptured bAVMs remain to be further clarified with future studies. Besides, the RCT study on brain AVMs is insufficient to date and urgently needed.

Although conservative treatment remains controversial, medication therapies are thought to be more promising than expectant therapy. Medication therapies take effects on pathophysiological processes of bAVMs to disturb their development, growth, and rupture (309). Three pathophysiological pathways have received the most in-depth investigations, including the overexpression of vascular endothelial growth factor (VEGF), impairment of Blood-brain barrier, and excessive activity of matrix metalloproteinases (MMPs). Bevacizumab, a humanized monoclonal anti-VEGF antibody which might decrease the hemorrhagic risk of unruptured bAVMs (310–316), and shorten the latency period of stereotactic radiosurgery (317, 318). Thalidomide and Lenalidomide, the immunomodulators acting on BBB impairments might reduce micro-hemorrhage in perinidual area (313, 319–321). Tetracyclines, the antibiotic targeting the MMPs pathway, might non-selectively to increase vascular stability by inhibit MMP-9 overexpression and decrease risks of spontaneous bleeding (322). Medications aiming at other pathways are under investigation in the early-stage as well, including MEK inhibitors engaged in KRAS mutations (323), angiotensins II receptor antagonist the regulator of BMP signaling pathway (324), and Notch inhibitors involved in its signaling pathway (325). Meanwhile, medications are considered effective on neuroprotection, including Glibenclamide, neuroglobin, and NA-1 (Tat-NR2B9c) (326–328). However, the side effects of targeted medications have to be considered (329, 330). Targeted medications for bAVM management remain to be further investigated.

## Author Contributions

MW, YJ, CZe, CZh, QH, WT, HQ, YY, and YC designed and conceptualized this work and participated in the literature review. MW, YJ, CZe, and CZh participated in drafting the manuscript. YC, WJ, and A-lL critically revised the specialized sections. HS, DZ, DK, SW, and JZ critically revised the manuscript in general for important intellectual content. All authors contributed to the article and approved the submitted version.

## Conflict of Interest

The authors declare that the research was conducted in the absence of any commercial or financial relationships that could be construed as a potential conflict of interest.
